# Successful prevention of extremely frequent and severe food anaphylaxis in three children by combined traditional Chinese medicine therapy

**DOI:** 10.1186/s13223-014-0066-5

**Published:** 2014-12-20

**Authors:** Lauren Lisann, Ying Song, Julie Wang, Paul Ehrlich, Anne Maitland, Xiu-Min Li

**Affiliations:** Division of Allergy and Immunology, Department of Pediatrics, Icahn School of Medicine at Mount Sinai, New York, NY 10029 USA; Medical Student at Icahn School of Medicine at Mount Sinai, New York, NY USA; Department of Pediatrics, New York University Langone Medical Center, New York, NY USA; Division of Allergy & Clinical Immunology, Icahn School of Medicine at Mount Sinai, New York, NY 10029 USA; Comprehensive Allergy & Asthma Care, PLLC, Eastchester, NY 10709 USA

**Keywords:** Traditional Chinese medicine, Food allergy, Food induced anaphylaxis

## Abstract

**Background:**

Despite strict avoidance, severely food-allergic children experience frequent and potentially severe food-induced anaphylaxis (FSFA). There are no accepted preventive interventions for FSFA. A Traditional Chinese Medicine (TCM) formula prevents anaphylaxis in murine food allergy models, and has immunomodulatory effects in humans. We analyzed the effects of TCM treatment on three pediatric patients with FSFA.

**Case description:**

Three FSFA patients (P) ages 9–16 years (P1 allergic to milk; P2 and P3 to tree nuts) qualified for case analysis. All experienced numerous reactions requiring administration of rescue medications and emergency room (ER) visits during the 2 years prior to starting TCM. P1 experienced approximately 100 reactions, 50 epinephrine administrations, 40 ER visits, and 3 admissions to intensive care units. P2 experienced 30 reactions, all requiring epinephrine administration, as well as 10 emergency hospitalizations. P3 experienced 400 reactions, five of which required epinephrine administration and ER visits. TCM treatment markedly reduced or eliminated reactions in all. P1 experienced no reactions after 2.5 years of TCM. P2 experienced no reactions after 1 year of TCM treatment, at which time she passed an oral almond food challenge. She continues to be reaction-free 6 months off TCM while consuming nuts. P3 has achieved a 94% reduction in reaction frequency following 7 months of TCM, has discontinued daily antihistamine use, and has required no epinephrine administrations or ER visits.

**Conclusions:**

Three children treated with TCM experienced dramatic reductions or elimination of FSFA. This regimen appears to present a potential option for FSFA, and warrants further investigation in controlled clinical studies.

**Electronic supplementary material:**

The online version of this article (doi:10.1186/s13223-014-0066-5) contains supplementary material, which is available to authorized users.

## Background

Food allergy is a leading cause of anaphylactic reactions treated in emergency departments in the United States [1,2], where it accounts for 31% of anaphylaxis cases [3], and is a growing public health concern. Anaphylaxis is an acute potentially fatal systemic type I hypersensitivity reaction [4]. The most cited working definition of food anaphylaxis (FA) was proposed by Sampson and colleagues in 2006 [4,5]. FA is acute onset of an illness with involvement of skin/mucosal tissue, airway compromise, and gastrointestinal symptoms, or reduced blood pressure and associated symptoms [1,5]. The most dangerous symptoms include breathing difficulties, a drop in blood pressure, and shock, which are potentially life-threatening. Given the increased awareness of food allergy by consumers and food manufacturers, frequency of accidental exposures appears to have been reduced [6,7]. A recent study reported that the annualized reaction rate in milk, egg and peanut allergic children was 0.81 per year [6]. However, extremely food-allergic children experience more frequent and potentially severe food-induced anaphylaxis (described herein as FSFA) despite attempted strict avoidance. Although the majority of food-induced reactions are triggered by ingestion; in extremely sensitive children severe reactions can also be triggered by inhalation [8,9] and skin contact [10,11]. Additional interventions that prevent frequent and potentially severe reactions while practicing food avoidance are urgently needed.

Traditional Chinese Medicine (TCM) is a medical system that primarily uses Chinese herbal medicines, acupuncture and acupressure. TCM has a long history of human use in China, Japan and Korea, and is beginning to play a role in the healthcare systems in the United States and other Western countries as a stand-alone or integrative practice. Unlike in Asia, the European Union and the UK [12], Chinese herbal medicines in the U.S are classified as dietary supplements [13]. In 2004, the US Food and Drug Administration (FDA) provided guidance for investigating botanical drug products including formulas comprised of multiple herbal constituents [14]. Although preclinical and clinical studies are limited, several suggest that TCM herbal formulas may have potential for treating food allergies [15,16]. Food Allergy Herbal Formula (FAHF)-1, FAHF-2, and butanol-extracted FAHF-2 (B-FAHF-2) (derived from the classical TCM formula *Wu Mei Wan*) prevent systemic anaphylaxis in murine models of food allergies [17-19]; and have been US FDA approved as botanical investigational new drugs. Phase I clinical studies showed that FAHF-2 is safe, had an immunomodulatory effect on T cells and suppressed basophil activation [20,21]. Herbal Formula-3 inhibited food allergy reactions in rats by stabilizing mast cells by modulating calcium mobilization [22]. The traditional Japanese herbal medicine Kakkonto suppressed the occurrence of allergic diarrhea and decreased the number of mast cells in the proximal colons in a murine food allergy model [23]. In addition, acupuncture has been reported to reduce wheal size following allergen skin tests and to reduce basophil activation in individuals with atopic dermatitis [24,25]. In addition, we investigated the effects of extracts of 70 TCM herbs on cultured human B cells, and found that several directly suppressed IgE synthesis [26]. Potential TCM effects on FSFA in children have not been previously investigated. This retrospective study of 3 pediatric patients with extraordinarily severe FSFA shows, for the first time, that a combined TCM therapy regimen prevented FSFA.

## Methods

### Inclusion criteria

Individuals selected for this report met the following criteria: physician (allergist) diagnosed severe food allergy (as documented by physician-determined history of allergic reactions to food allergen, a positive skin test result and/or elevated food allergen specific IgE level), clinical reactions that became worse over time prior to TCM treatment, and experienced FSFA including multiple life-threatening episodes (>10 reactions and >2 epinephrine uses in the 3 months prior to starting TCM therapy), adherence to the TCM regimen, and had completed at least 6 months of TCM therapy.

### Exclusion criteria

FSFA patients concurrently treated with omalizumab and TCM.

### Laboratory tests

Since each patient had a unique clinical situation, this information was included within the description of each case.

### TCM therapy

TCM therapy consisting of *Modified Pruni Mume Formula* (Remedy A), *Fructus Jujubae Formula* (Remedy B), a *Phellodendron chinensis* (*P. chinesis*) containing herbal bath additive (Remedy C), and herbal cream (Remedy D) (Additional file 1: Herbal constituents and doses). Remedies A and B were used as dietary supplements. Use of additional herbs is noted in each case discussion when applicable. Acupuncture/acupressure was also administered during TCM clinic visits. Patients were seen at the Ming Qi Natural Health Care Center, Integrative Health and Acupuncture in New York City.

### Visits

All patients had to have at least one initial clinical visit and completed 6 months of TCM treatment. Since the treatment courses of individual cases varied, the number of revisits varied and is described in each case.

### Safety and efficacy parameter

**Figure 1:**
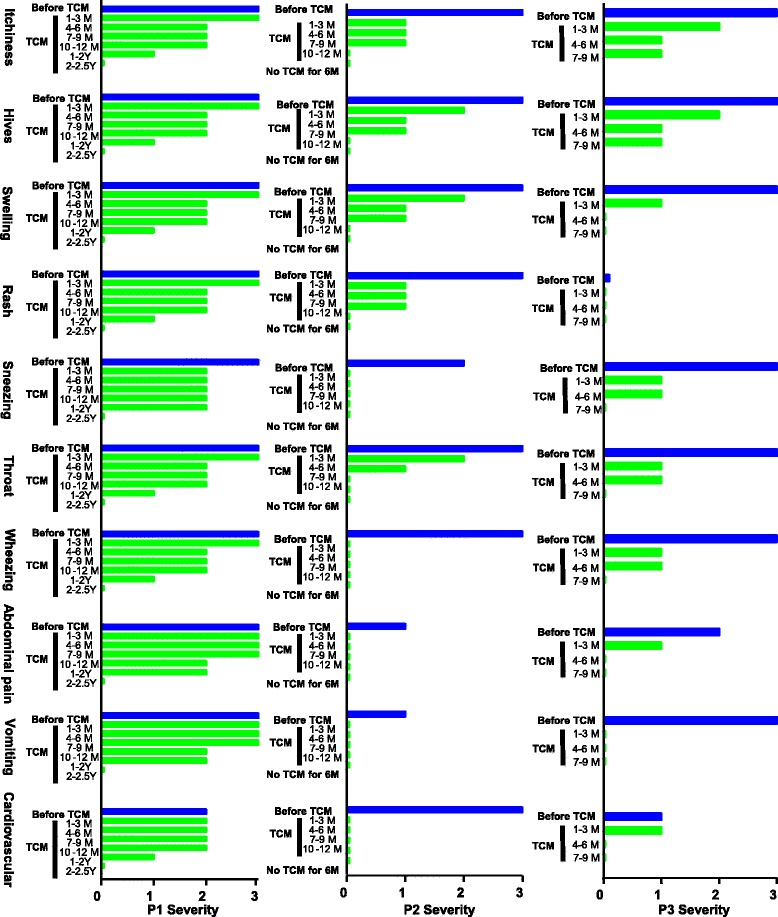
**Symptom scores before, during and after TCM treatment.** Severity of symptoms, during the previous 3 month period and during and post TCM therapy at different times as indicated were recorded in a patient history form and patient files. Blue bars indicate reaction scores during the 3 months before TCM. Green bars indicate symptom scores during and after TCM as indicated. All patients experienced reductions in symptom severity over the course of TCM. Lisann et al.

**Figure 2:**
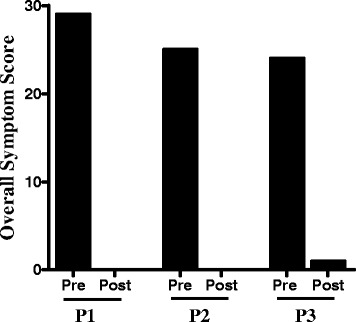
Total symptom scores before and after TCM treatment. Total symptom scores of reactions were calculated during the 3 month period before receiving TCM treatment and the last 3–6 months period of TCM therapy based on the data shown in Figure 1.

Safety and tolerability is defined by no TCM remedy related reactions, and no abnormal liver and kidney function test result (aspartate amino transferase (AST), alanine amino transferase (ALT) and blood urea nitrogen (BUN) levels when data were available. The efficacy parameter was based on prevention of frequent reactions, reduced medication use and ER visits (Table 1), reduction of individual (Figure 1), and total symptom scores (Figure 2), using a modified version of the Grading of Food-Induced Anaphylaxis [27] (Additional file 1: Table S1). Data were obtained from patient history forms and interviews before, during, and after the TCM treatment course of February 2011 through February 2014. Data were collected every 3 months during the first year, and every 6 months after one year of TCM treatment. In addition, Quality of Life/Distress over time (using a modified version of the “Food hypersensitivity family impact (FLIP) questionnaire [28] (Additional file 1: Table S2) were also evaluated.

**Table 1:** **Frequency of reaction, medication use and ER visits before, during and after TCM therapy**

**# of uses or occurrences**	**2 year period before TCM**			**During and after the courses of TCM**						
	**Total**	**Ave./Year**	**Prior 3 M**	**1-3 M**	**4-6 M**	**7-9 M**	**10 – 12 M**	**1 - 1.5 Y**	**1.5- 2 Y**	**2 - 2.5 Y**
Patient 1										
Allergic Reactions	100	50	10	8	3	3	2	1	1	0
Epinephrine	50	25	5	3	1	1	1	0	0	0
Diphenhydramine (p.r.n.)	100	50	10	8	3	3	2	1	1	0
Prednisone	50	25	5	5	1	1	1	0	0	0
ER visits	40	20	5	0	0	0	0	0	0	0
Patient 2										
Allergic Reactions	30	15	10	1	2	1	0	0	0**	ND
Epinephrine	34	17	10	0	0	0	0	0	0^**^	ND
Cetirizine (daily)	730	365	93	30	0	0	0	0	0^**^	ND
Cetirizine (p.r.n.)	30	15	10	1	0	0	0	0	0^**^	ND
Diphenhydramine (daily)	730	365	93	30	0	0	0	0	0^**^	ND
Diphenhydramine (p.r.n.)	30	15	10	1	1	1	0	0	0^**^	ND
Prednisone	2	NA*	1	0	0	0	0	0	0^**^	ND
ER visits	10	5	5	0	0	0	0	0	0^**^	ND
Patient 3										
Allergic Reactions	400	200	45	10	2	1	ND	ND	ND	ND
Epinephrine	5	2.5	2	1	0	0	ND	ND	ND	ND
Diphenhydramine (p.r.n.)	400	200	45	10	2	1	ND	ND	ND	ND
Levocetirizine (daily)	730	365	90	60	30	0	ND	ND	ND	ND
Fexofenadine (daily)	730	365	90	60	0	0	ND	ND	ND	ND
Prednisone	5	2.5	0	1	0	0	ND	ND	ND	ND
ER visits	5	2.5	1	1	0	0	ND	ND	ND	ND

Reactions, medication use and ER visits during the prior 2-year period history and during treatment were documented in patient history forms. Patient 1 has completed 2.5 years of TCM treatment. She experienced no reactions during the last 6 months of her TCM course, but continues TCM treatment because she has not yet undergone a food allergy challenge. Patient 2 stopped TCM after 1 year, due to absence of reactions and a successful food allergy challenge. Patient 3 has completed 7 months of TCM, and has experienced a marked reduction in food allergy reactions. He continues TCM therapy. Daily, daily use as preventive treatment. p.r.n., use as needed. *NA, not applicable, because P2 had prednisone reactions and had to stop prednisone use in the subsequent treatment of anaphylactic reaction. **6 months after stopping TCM. ND, not done.

## Case presentation

### CASE 1

Patient 1 (P1), a 13-year old female with physician-diagnosed severe milk allergy began TCM in February 11, 2011 with the goal of preventing reactions while continuing on a dairy restricted diet.

#### Pre TCM

**Figure 3:**
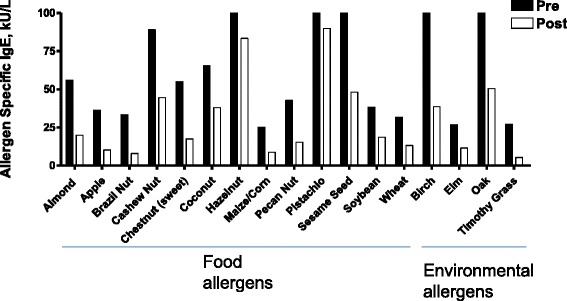
**P3 antigen specific-IgE levels before and after TCM.** Prior to the initial TCM clinic P3 underwent extensive IgE testing by his allergist which revealed poly-sensitization to multiple food and environmental allergens. Seventeen food and environmental allergen-specific IgE levels were above 17.6 kU/ L. His pre-TCM IgE levels >17.6 kU/L were all reduced by TCM therapy.

This patient was diagnosed with milk allergy at 3 months of age after an ER visit due to anaphylaxis (widespread hives, vomiting and difficulty breathing) immediately following ingestion of cow milk based formula. She subsequently experienced numerous reactions; and the frequency and severity of these reactions worsened over time. Despite a dairy restricted diet, during the 2-year period prior to starting TCM she experienced >100 reactions (at times a reaction every few days, averaging 50 reactions/year). Fifty events required prednisone and epinephrine administration, 40 required ER visits, and 3 resulted in intensive care unit (ICU) admissions. In the three months before TCM, her reactions became even more severe; 5 of 10 events required epinephrine and ER visits. These reactions were characterized by hives, rash, lip swelling, throat tightness, wheezing, stomach pain, headache, weakness, dizziness, syncope, hypoxia, and drop in blood pressure. Her overall severity score during the 3-month period before beginning TCM was 29 (Figures 1 and 3). Her reactions were triggered by inhalation and contact in addition to accidental ingestion of trace amounts of dairy products. Anaphylaxis events were so frequent and severe that she was unable to attend school in the 4 months prior to her first TCM visit. She also complained of chronic stomach discomfort. Her milk protein specific IgE levels were elevated at 37.3 kU/L. She did not undergo milk protein skin testing because her history of severe skin contact induced reactions, raised concern that severe reactions might occur.

#### During the course of TCM

This patient lives in another state that outside New York state. The impact of these reactions on quality of life is illustrated by the fact that the patient’s father drove for three days to bring her to New York City for the first TCM visit because of fear of a possible reaction to trace amounts of milk protein while on an airliner. She was prescribed all 4 TCM remedies: Remedy A, started with 1 pill, gradually increasing to 5 pills, 10 pills and 12 pills, twice daily (b.i.d), and then three times daily (t.i.d.). 12 capsules t.i.d was the full dose for patients 12 and above based on the manufacturing extraction yield of raw herbs (Additional file 1: Herbal constituents and doses). The protocol to gradually increase the dose was to ensure the safety and tolerability. Remedy B, 3 pills, b.i.d.; Remedy C (bath additive) 2 packs per bath once a day (q.d.), and Remedy D (cream) applied to her entire body, including the face, q.d.. Since her reactions often began with throat tightness or pain, *Fructus Arctii Lappae*, which has been traditionally used for throat ailments [29], was added during the treatment course. Acupuncture was also performed at each visit. She tolerated the medicine regimen very well and after approximately 3 months no longer complained of stomach discomfort that she used to have before TCM. Follow-up visits took place every 3–6 months, a total 8 visits. Her FSFA slightly improved after 6 months of TCM and greatly improved after 1.5 years of TCM, and she was able to resume school. After 2 years of treatment, the number of reactions was reduced by 90%. None required epinephrine, or ER visit. Symptom severity was reduced by 56%, based on total scores (Figures 1 and 3). She was able to work a summer job as a lifeguard, and she and her father no longer feared flying to New York for her two-year TCM visit. She experienced no reactions during the final 6 months of TCM (Years 2–2.5) (Table 1, Figures 1 and 3). Her baseline milk specific IgE level of 37.3kU/L has been declined to 19.0kU/L. Her FLIP score reduced to 25 from 35. No abnormal liver and kidney function test results before or after TCM therapy (AST, 20 and 18, reference 14-37u/L; ALT, 16 and 30, reference 8–36 U/L; BUN, 12 and 9, reference 7–25 mg/dL) were observed. She has reached her primary goal of avoiding reactions while on a dairy restricted diet, and is continuing TCM therapy with a new goal of maintaining the beneficial effect and to possibly become tolerant to milk allergens.

### CASE 2

P2 is a 16 year-old female with physician diagnosed severe tree nut allergy who began TCM therapy in June 20, 2012 with the goal of reducing her incidence of tree nut-induced anaphylaxis.

#### Pre TCM

She was first diagnosed with severe nut allergy at age 13 after an ER visit due to anaphylaxis triggered by eating nut containing foods in a Thai restaurant, exhibited as widespread hives, lip swelling and vomiting, followed by throat tightness and difficulty breathing. Despite removing nuts from her diet, during the 2 years prior to starting TCM she experienced 30 severe reactions (average 15 reactions/year) requiring 34 epinephrine and 30 additional diphenhydramine doses (she had been on daily diphenhydramine and cetirizine during the 2 year period), and 10 emergency hospital admissions (Table 1). One episode required 4 epinephrine doses prior to emergency hospital admission following helicopter transport. She was most sensitive to almonds, and vomited and developed wide spread hives after skin prick testing with almond 2 years prior to TCM. P2 has blood/needle phobias. She did not undergo blood tests for specific IgEs against nuts. During the year prior to initiating TCM, her reactions progressed much more rapidly from hives and vomiting to hypoxia and circulatory collapse. She was severely sensitive to trace nut allergen exposure by inhalation and contact as well as by ingestion. Her symptom score was 25 during the 3 months prior to initiating TCM (Figures 1 and 3). She missed approximately 50% of school days and had to withdraw from her school sports team. As a consequence, she developed anxiety/depression and was on sertraline during the 2 months prior to beginning TCM. Her mother (an ER physician) arranged numerous consultations with allergists. They were subsequently told by an allergist that there was nothing more to be done, and there was nothing on the horizon that would help her severe nut anaphylaxis. After the mother read publications showing that TCM herbal medicines abolished food anaphylaxis in murine models, they sought similar therapy.

#### During and after TCM

This patient followed the same initial protocol as P1. However, unlike P1, she responded relatively quickly; experiencing only one reaction during the first 3 months of treatment (moderate hives, face swelling, and throat pain, but no gastrointestinal symptoms, wheezing, cardiovascular, or neurological symptoms), which was treated with additional diphenhydramine. She was maintained on 10 pills Remedy A b.i.d. Her anxiety also dramatically improved and she discontinued sertraline after 2 months of TCM. After 4–6 months of TCM, she experienced two mild reactions. The first included mild hives, rash, and lip swelling only, and was treated with additional diphenhydramine. The second reaction consisting of a few hives, did not require additional diphenhydramine. After 6 months, she discontinued daily anti-histamine use (Table 1 P2). She experienced no reactions during the final 3 months (months 10–12) of treatment. Total symptom severity score declined from 25 to 0 (Figures 1 and 3). Since she lives in another country, her mother had monthly phone consultations for the first 2 months, and then every 2–3 months plus email communication until completing one year of TCM therapy, at which time she passed a nut challenge. The tentative 12-month visit was cancelled. Her mother continued to communicate by emails up to 6 months after challenge. During this period she accidentally consumed nut-containing pastry and experienced no allergic reaction. She subsequently passed challenges with almond and mixed nuts. Importantly, she remains free of allergy symptoms while consuming tree nuts *ad libitum* 6 months off TCM. Her FLIP score was reduced from 39 to 0. This patient achieved her primary goal. Laboratory data are not available.

### CASE 3

Patient 3 is a 9 year-old male with allergist diagnosed severe tree nut and fruit allergies who began TCM therapy in June 2013 with the goal of reducing the frequency of his reactions.

#### Pre TCM therapy

This patient was diagnosed with nut allergy at 7 years of age after an ER visit due to anaphylaxis (projectile vomiting, inability to move his legs due to cramps, throat closed, diffuse hives and breathing difficulty) after ingesting chocolate containing mixed tree nuts. Despite an all nut restricted diet, his reactions became more frequent and severe. He also developed reactions to additional foods, including coconut, sesame seed, certain fruits and wheat, which he had previously tolerated. Reactions were triggered by inhalation and contact as well as accidental ingestion of trace amounts of offending foods. During the prior 2 year period he experienced approximately 400 reactions (every few days and at times daily, averaging 200 reactions per year) while on daily levocetirizine. His allergist added daily fexofenadine because levocetirizine alone failed to reduce the number or severity of reactions during the prior 6 months. However, his frequent reactions did not decrease. His most common reactions included widespread itching, diffuse hives, swollen tongue, projectile vomiting, and throat tightness. The most severe reactions included wheezing, hypoxia, muscle cramping, weakness, and increased heart rate. Five reactions required epinephrine and ER visits. He had positive skin test results to almond, pistachio, sesame, peanut, soy bean, coconut, apple and wheat 6 months prior to TCM. Extensive serum IgE testing ordered by his primary allergist 2 years and one month prior to his initial TCM visit revealed that he was poly sensitized to multiple food and environmental allergens. His IgE levels to 65 food and environmental allergens were elevated. Seventeen were above 17.6 KU/L (classified as very high), 13 of these were to food allergens (Figure 3). He had a history of reactions to all 13 foods, but was most sensitive to almonds. He also complained of chronic stomach discomfort and headaches, and had developed a sleep disorder approximately 6 months before beginning TCM treatment.

### During TCM

This patient underwent the same regimen as P1 and P2. However, the maximum dose of Remedy A was 8 pills, b.i.d (adjusted based on his age, approximately 1/3 of full dose). He also received monthly acupuncture. The frequency and severity of his reactions decreased by 50% during the first 3 months of TCM (from 20 reactions per month at baseline to 10 per month at 3 months of TCM). During the 4–6 months treatment period, he experienced only two mild reactions. He has discontinued fexofenadine. After 7 months of TCM, he also discontinued levocetirizine, and has experienced only one mild reaction treated with diphenhydramine. His overall symptom score declined from 24 to 1 (94% reduction, Figure 2). All 17 foods and environmental allergen-specific IgE levels, previously above 17.6 kU/L, were reduced (Figure 3). His liver and kidney function test results were all within the normal range before and after TCM (AST, 26 and 23 U/L, reference 12-32U/L; ALT 15 and 18 U/L, reference 8–30 U/L; BUN, 11 and 8, reference 7–20 mg/dL). His FLIP score was reduced from 37 to 22. In addition, after the first 3 months of treatment, he no longer experienced stomach discomfort or headaches; and he no longer has a sleep disorder. He reached his primary goal, and is continuing TCM therapy to maintain the efficacy and perhaps to induce a tolerance to some of the foods.

## Discussion

This case study describes 3 FSFA patients who underwent TCM therapy with the goal of reducing the frequency and severity of their food induced anaphylactic reactions. In all cases, food allergy was diagnosed after ER visits due to anaphylactic reactions. Their FSFA generated significant stress for their families. P1 anaphylaxis episodes began in infancy, and increased in frequency and severity over time. P2 had a 3 year, and P3 had a 2 year period of anaphylaxis episodes that also increased in frequency and severity over time prior to TCM therapy. We therefore analyzed their reaction history during the 2-year period prior to starting TCM. We also conducted a separate analysis of frequency and severity of their reactions during the 3-month period immediately prior to starting TCM. All reported extraordinarily frequent and severe reactions during the 2-year pre-TCM period. Attempts to avoid ingestion of offending foods were not sufficient, because anaphylaxis in these patients could also be triggered by inhalation and skin exposure. The number of accidental reactions in these patients appeared to be 15–100 times higher than the recently reported rate of 0.8 reactions annually [6]. This might be due to their extreme sensitivity to minute quantities of non-ingested allergen. All families denied that any episode was associated with acute stress, ruling out isolated panic disorder as responsible for the physiologic phenomena. Although the majority of clearly defined food induced reactions are triggered by ingestion, anaphylaxis due to non-ingested food allergen exposure has also been reported. For example, Vitaliti et al. [8] described a child who developed anaphylaxis after inhaling lentil vapors. Tan et al. [10] reported severe food allergy reactions following skin contact and inhalation of milk, egg or peanut in 5 children. Liccardi et al. [11] reported a severe systemic allergic reaction in a 16-year-old boy induced by accidental skin contact with cow milk. A recent large cohort study reported that although 80.7% of food allergy reactions were triggered by ingestion, 12.9% were triggered by skin contact, and 1.2% by inhalation [6]. Although the extremely severe FSFA in the patients described in the present report represents only a fraction of food allergy patients; this group of patients is most in need of therapy. All families expressed frustration about the lack of understanding by others, especially health care providers, regarding how sensitive their children were to food allergens.

This study is the first to demonstrate that comprehensive TCM therapy can prevent or markedly reduce FSFA and reduce antigen IgE levels. Mast cell and basophil IgE sensitization and subsequent activation following relevant antigen exposure are the key triggers of clinical anaphylactic reactions. Because FSFA clinical expression involves multiple organ systems, a combination of 4 TCM remedies was used for all 3 patients. The medicinal properties of herbs in each formulation have been studied in other contexts. Remedy A contains the 8 herbs in *Wu Mei Wan* (from which FAHF-1 and FAHF-2 were developed), which originated from the classical herbal formula described in the book “Shang Han Lun” by Chinese physician Zhang Zhong-Jing (150 AD) used to treat gastrointestinal conditions and anaphylactic-like symptoms [30]. FAHF-2 prevents anaphylaxis in animal model of food allergy, and suppresses IgE synthesis and mast cell and basophil activation in animal models [17] and human basophil activation *ex vivo*. Remedy A differs from FAHF-2 by not containing *Coptis chinesis* (*C. chinesis*) but instead a 50% higher dose of *P. chinesis* extract than that of *C. chinesis* in FAHF-2 [31]. Both herbs are used similarly in TCM practice. In recent years, *C. chinesis* has become increasingly expensive (7.5 times more expensive than *P. chinesis*). Importantly *P. chinesis* extract has been shown to inhibit IgE production in vitro [26]. Remedy B, used to alleviate patients’ gastrointestinal symptoms, containing 6 herbs is based on modified *Xiang Sha Yang Wei Wan*, the classical herbal formula described in the TCM book “Wan Bin Hui Chun” by Chinese physician Lon Tin Xian (1587 AD), which is used to reduce stomach ache and indigestion [30]. In addition, since all patients were highly sensitive to food allergen skin contact, and since *P. chinesis* compounds inhibit mast cell degranulation [17] and IgE production, a *P. chinesis* containing bath additive (Remedy C), and a cream (Remedy D) used to treat eczema [32] were also utilized. Although all 3 patients had a remarkable response to TCM therapy, P1 required longer treatment than P2 and P3 before clinical improvement was evident. P1 experienced no reactions only during the final 6 months of a 2.5 year treatment period. P2 and P3 showed more rapid improvement, which became evident in the first 3 months of TCM therapy. Of most significance is that P2 reestablished full tolerance to almonds and other tree nuts, and continues to tolerate all nuts 6 months off TCM. In parallel with FSFA improvement, all patients’ chronic stomach discomfort resolved after approximately 3 months of TCM, and has not reoccurred. Their social activity and quality of life are improved: P1 resumed school and took a summer job, P2 resumed school sports and discontinued sertraline, and P3 no longer has a sleep disorder. Their FLIP scores are also reduced, most dramatically in P2 (from 39 to 0). In addition to the reported subjective clinical information, laboratory data were obtained from P1 and P3. These data showed that TCM therapy reduced both patients’ IgE levels, which is consistent with findings in animal food allergy models [19], a cultured human B cell line [26], and human PBMCs from food allergic children [33].

Adherence to this type of combined TCM therapy is challenging because patients are required to adhere to a protocol that includes consuming many pills daily, as well as external treatment regimens. In contrast to a recent report showing that subjects exhibited poor adherence to a treatment protocol requiring consumption of a large number of pills in a phase II trial [34], the patients in this study strictly followed the TCM protocol (documented by parents). Their motivation may be due to their extreme sensitivity and history of frequent reactions. We do not know if all remedies used by these patients were essential, and further study would be required to determine their individual effects.

Diagnosis of IgE-mediated food allergy requires evidence of both sensitization and clinical symptoms after exposure to the allergen. Either skin-prick testing or measurement of specific IgE levels is recommended for identifying foods that may provoke IgE-mediated allergic reactions [35]. P1 has evidence of milk IgE sensitization and numerous well defined reactions to milk products by inhalation and skin contact, in addition to accidental ingestion. Although IgE testing was not performed, P2 exhibited systemic reactions (hives and vomiting) following skin testing with mixed tree nuts. This together with her history of well defined episodes of anaphylaxis made P2 qualified for this case review based on our FSFA criteria. Patient 3 had many positive skin test results and positive IgE levels. He also had a history of reactions to at least 13 foods before beginning TCM as illustrated in Figure 3.

Although it is widely known that mast cells are activated in the context of an allergic reaction by allergen-induced cross-linking of surface IgE/FcεRI (the high-affinity receptor for the Fc region of IgE), it should be recognized that many other stimuli and conditions can cause mast cell activation and result in anaphylaxis [36]. Frequent anaphylaxis in a patient should prompt a clinician to include mast cell disorders in the differential diagnosis. Mast cell activation disorder (MCAD) has been distinguished into 2 major forms, clonal and nonclonal. Systemic mastocytosis (SM) and monoclonal mast cell activation disorder (MMAS), common forms of primary MCAD, implicate dysregulation of C-kit gene in a clonal population of mast cells, leading to increased physical burden of mast cells [36]. Skin lesions (eg, urticaria pigmentosa), recurrent unexplained anaphylaxis, and unexplained cytopenias are clinical characteristics [36]. Non-clonal mast cell activation syndrome (nc-MCAS, MACS) is a common form of idiopathic MCAD [36,37]. Factors extrinsic to mast cells lead to recurrent, inappropriate release of mast cell mediators, including histamine and lipid mediators. The differential expression and release mediators of this multifaceted innate immune cell population leads to different hypersensitivity symptoms –chronic urticaria, pruritis, flushing, gastrointestinal distress (nausea, vomiting, abdominal pain, cramping, bloating), respiratory symptoms, cardiovascular compromise, and cognitive impairment such as poor concentration and memory and brain fog. Both primary and idiopathic MCAD usually have no objective evidence of food specific IgE allergy by ImmunoCAP® and percutaneous testing, which distinguishes them from IgE-mediated hypersensitivity reactions, a form of secondary MCAD [36,37]. All 3 cases reported in this study had elevation of IgE and/or positive skin test to specific foods as well as well defined specific food exposure-induced reactions, which is in agreement with food induced anaphylaxis [35]. In addition, none of these individuals exhibited urticaria pigmentosa, chronic urticaria, pruritis, flushing, stomach bloating, or brain fog. Thus MMAS or MACS was not entertained in these cases.

Patient 1 had severe symptoms as a result of cow's milk formula at 3 months of age. Food protein–induced enterocolitis syndrome (FPIES) is in the differential diagnosis for this age group. FPIES has a unique clinical expression that distinguishes it from IgE mediated food anaphylaxis. Acute FPIES manifests as profuse repetitive vomiting and lethargy, typically occurring 1–3 hours after ingestion of the offending allergen, and occasionally followed by diarrhea several hours later [38,39]. 90% of children outgrew FPIES by 3 years of age. Diagnosis is primarily based on history because specific IgE and skin prick tests are typically negative [39-41]. Furthermore, FPIES reactions do not involve skin [42]. P1 developed widespread hives, projectile vomiting, and difficulty breathing within minutes following ingestion of milk-based formula. It is highly unlikely that these symptoms would lead to a diagnosis of FPIES at time of presentation. This patient subsequently tested positive for milk specific IgE. Her milk allergy persisted to adolescence and became worse 2 years prior to TCM therapy despite attempted dairy product avoidance, and her reactions were often trigged by skin contact and inhalation of trace amounts of milk allergen. Her reactions were acute, starting with hives and progressing rapidly to respiratory reactions; in most severe events, cardiovascular changes were also seen. Overall, given the acute reactions, elevation of IgE, clinical expression and persistence of milk allergy, and skin and inhalation induced reactions to trace levels of milk protein, FPIES has not been considered.

Limitations of this study include the limited number of cases, possible recall bias and lack of knowledge of mechanisms underlying clinical effects. Patients did not undergo diagnostic food challenge because of fear of severe reactions, their convincing history of reactions, rescue medication use and ER visits. Food challenge is not necessary and may pose a potential risk in patients with FSFA. This is in agreement with previous publications [10,27].

## Conclusions

We observed 3 pediatric patients with extraordinarily frequent and potentially life threatening severe food-induced anaphylaxis, and found that combined TCM therapy successfully prevented FSFA, thereby improving the quality of life of patients and their families. Patients who experience frequent and potentially fatal food-induced anaphylaxis have severely limited capacity to participate in activities of “normal” daily life. Many of these patients have exhausted all of their options and see no chance of improvement through conventional treatments. The therapy presented in the current case reports presents a potential option for improvement in these patients. Prospective clinical studies and randomized clinical trials that will further determine the effectiveness in a large group of patients and assess immunological changes are warranted.

## Consent

Since this study was conducted in the format of a case review and the number of cases was below the 4 case limit, Institutional Review Board approval was not required. All patients gave written informed consent for TCM therapy at the initial clinic visit.

## Additional file

Additional file 1:
**Supplemental information.**

## Abbreviations

FSFA: Frequent and severe food-induced anaphylaxis
TCM: Traditional Chinese medicine
FAHF: Food allergy herbal formula
B-FAHF: Butanol-extracted FAHF

## References

[1] Sampson HA (2003). Anaphylaxis and emergency treatment. Pediatrics.

[2] Vetander M, Helander D, Flodstrom C, Ostblom E, Alfven T, Ly DH, Hedlin G, Nilsson C, Wickman M (2012). Anaphylaxis and reactions to foods in children–a population-based case study of emergency department visits. Clin Exp Allergy.

[3] Wood RA, Camargo CA, Lieberman P, Sampson HA, Schwartz LB, Zitt M, Collins C, Tringale M, Wilkinson M, Boyle J, Simons Fe (2014). Anaphylaxis in America: the prevalence and characteristics of anaphylaxis in the United States. J Allergy Clin Immunol.

[4] Panesar SS, Javad S, de Silva D, Nwaru BI, Hickstein L, Muraro A, Roberts G, Worm M, Bilò MB, Cardona V, Dubois AE, Dunn Galvin A, Eigenmann P, Fernandez-Rivas M, Halken S, Lack G, Niggemann B, Santos AF, Vlieg-Boerstra BJ, Zolkipli ZQ, Sheikh A, EAACI Food Allergy and Anaphylaxis Group (2013). The epidemiology of anaphylaxis in Europe: a systematic review. Allergy.

[5] Sampson HA, Munoz-Furlong A, Campbell RL, Adkinson NF, Bock SA, Branum A, Brown SG, Camargo CA, Cydulka R, Galli SJ, Gidudu J, Gruchalla RS, Harlor AD, Hepner DL, Lewis LM, Lieberman PL, Metcalfe DD, O'Connor R, Muraro A, Rudman A, Schmitt C, Scherrer D, Simons FE, Thomas S, Wood JP, Decker WW (2006). Second symposium on the definition and management of anaphylaxis: summary report–Second National Institute of Allergy and Infectious Disease/Food Allergy and Anaphylaxis Network symposium. J Allergy Clin Immunol.

[6] Fleischer DM, Perry TT, Atkins D, Wood RA, Burks AW, Jones SM, Henning AK, Stablein D, Sampson HA, Sicherer SH (2012). Allergic reactions to foods in preschool-aged children in a prospective observational food allergy study. Pediatrics.

[7] Sicherer SH, Burks AW, Sampson HA (1998). Clinical features of acute allergic reactions to peanut and tree nuts in children. Pediatrics.

[8] Vitaliti G, Morselli I, Di SV, Lanzafame A, La Rosa M, Leonardi S (2012). Urticaria and anaphylaxis in a child after inhalation of lentil vapours: a case report and literature review. Ital J Pediatr.

[9] Leonardi S, Pecoraro R, Filippelli M, Miraglia DG, Marseglia G, Salpietro C, Arrigo T, Stringari G, Ricò S, La Rosa M, Caffarelli C (2014). Allergic reactions to foods by inhalation in children. Allergy Asthma Proc.

[10] Tan BM, Sher MR, Good RA, Bahna SL (2001). Severe food allergies by skin contact. Ann Allergy Asthma Immunol.

[11] Liccardi G, De Falco F, Gilder JA, D'Amato M, D'Amato G (2004). Severe systemic allergic reaction induced by accidental skin contact with cow milk in a 16-year-old boy. A case report. J Investig Allergol Clin Immunol.

[12] **Herbal medicines regulation** http://www.mhra.gov.uk/Howweregulate/Medicines/Herbalmedicinesregulation/

[13] Dietary Supplement Health and Education Act of 1994. Public Law 103–417 National Institutes of Health, Office of Dietary Supplement Web site Accessed at http://ods.od.nih.gov/About/DSHEA_Wording.aspx

[14] The US Food and Drug Administration (FDA), Center for Drug Evaluation and Research. Guidance for Industry Botanical Drug Products. Revised ed. 2004

[15] Wisniewski JA, Li XM (2012). Alternative and complementary treatment for food allergy. Immunol Allergy Clin N Am.

[16] Li XM (2011). Treatment of asthma and food allergy with herbal interventions from traditional chinese medicine. Mt Sinai J Med.

[17] Song Y, Qu C, Srivastava K, Yang N, Busse P, Zhao W, XM Li (2010). Food allergy herbal formula 2 protection against peanut anaphylactic reaction is via inhibition of mast cells and basophils. J Allergy Clin Immunol.

[18] Srivastava K, Yang N, Chen Y, Lopez-Exposito I, Song Y, Goldfarb J, Zhan J, Sampson H, Li XM (2011). Efficacy, safety and immunological actions of butanol-extracted Food Allergy Herbal Formula-2 on peanut anaphylaxis. Clin Exp Allergy.

[19] Srivastava KD, Qu C, Zhang T, Goldfarb J, Sampson HA, Li XM (2009). Food Allergy Herbal Formula-2 silences peanut-induced anaphylaxis for a prolonged posttreatment period via IFN-gamma-producing CD8+ T cells. J Allergy Clin Immunol.

[20] Patil SP, Wang J, Song Y, Noone S, Yang N, Wallenstein S, Sampson HA, Li XM (2011). Clinical safety of Food Allergy Herbal Formula-2 (FAHF-2) and inhibitory effect on basophils from patients with food allergy: Extended phase I study. J Allergy Clin Immunol.

[21] Wang J, Patil S, Yang N, Ko J, Lee J, Noone S, Sampson HA, XM Li (2010). Safety, tolerability, and immunologic effects of a food allergy herbal formula (FAHF-2) in food allergic individuals: a randomized, double-blinded, placebo-controlled, dose escalation phase I study. Ann Allergy Asthma Immunol.

[22] Yang B, Li J, Liu X, Ma L, Deng L, Liu J, Liu Z, Ji Q (2013). Herbal Formula-3 inhibits food allergy in rats by stabilizing mast cells through modulating calcium mobilization. Int Immunopharmacol.

[23] Yamamoto T, Fujiwara K, Yoshida M, Kageyama-Yahara N, Kuramoto H, Shibahara N, Kadowaki M (2009). Therapeutic effect of kakkonto in a mouse model of food allergy with gastrointestinal symptoms. Int Arch Allergy Immunol.

[24] Pfab F, Huss-Marp J, Gatti A, Fuqin J, Athanasiadis GI, Irnich D, Raap U, Schober W, Behrendt H, Ring J, Darsow U (2010). Influence of acupuncture on type I hypersensitivity itch and the wheal and flare response in adults with atopic eczema - a blinded, randomized, placebo-controlled, crossover trial. Allergy.

[25] Pfab F, Athanasiadis GI, Huss-Marp J, Fuqin J, Heuser B, Cifuentes L, Brockow K, Schober W, Konstantinow A, Irnich D, Behrendt H, Ring J, Ollert M (2011). Effect of acupuncture on allergen-induced basophil activation in patients with atopic eczema:a pilot trial. J Altern Complement Med.

[26] Lopez-Exposito I, Castillo A, Yang N, Liang B, Li XM (2011). Chinese herbal extracts of Rubia cordifolia and Dianthus superbus suppress IgE production and prevent peanut-induced anaphylaxis. Chin Med.

[27] Sampson HA, Gerth VW, Bindslev-Jensen C, Sicherer S, Teuber SS, Burks AW, Dubois AE, Beyer K, Eigenmann PA, Spergel JM, Werfel T, Chinchilli VM (2012). Standardizing double-blind, placebo-controlled oral food challenges: American Academy of Allergy, Asthma and; Immunology-European Academy of Allergy and Clinical Immunology PRACTALL consensus report. J Allergy Clin Immunol.

[28] Mikkelsen A, Borres MP, Bjorkelund C, Lissner L, Oxelmark L (2013). The food hypersensitivity family impact (FLIP) questionnaire - development and first results. Pediatr Allergy Immunol.

[29] Bensky DCS, Stoger E (2004). Chinese herbal medicine: material and Meidica.

[30] Bensky D, Barolet R (1990). Chinese herbal medicine: formulas & strategies.

[31] Kattan JD, Srivastava KD, Zou ZM, Goldfarb J, Sampson HA, Li XM (2008). Pharmacological and immunological effects of individual herbs in the Food Allergy Herbal Formula-2 (FAHF-2) on peanut allergy. Phytother Res.

[32] Wisniewski J, Oh M, Nowak-Wegrzyn A, Steenburgh-Thanik E, Sampson H, Li XM (2010). Efficacy and safety of traditional chinese medicine for treatment of atopic dermatitis (AD). J Allergy Clin Immunol (Abstract).

[33] Yang N, Wang J, Liu C, Song Y, Zhang S, Zi J, Zhan J, Masilamani M, Cox A, Nowak-Wegrzyn A, Sampson H, Li XM (2014). Berberine and limonin suppress IgE production by human B cells and peripheral blood mononuclear cells from food-allergic patients. Ann Allergy Asthma Immunol.

[34] Ross J, Carlisle SK, Vazquez M, Jones SM, Pongracic J, Wang J (2014). Food Allergy Herbal Formula-2 (FAHF-2) – Adherence to treatment. J Allergy Clin Immunol (Abstract).

[35] Burks AW, Jones SM, Boyce JA, Sicherer SH, Wood RA, Assa'ad A, Sampson HA (2011). NIAID-sponsored 2010 guidelines for managing food allergy: applications in the pediatric population. Pediatrics.

[36] Picard M, Giavina-Bianchi P, Mezzano V, Castells M (2013). Expanding spectrum of mast cell activation disorders: monoclonal and idiopathic mast cell activation syndromes. Clin Ther.

[37] Akin C, Valent P, Metcalfe DD (2010). Mast cell activation syndrome: proposed diagnostic criteria. J Allergy Clin Immunol.

[38] Jarvinen KM, Nowak-Wegrzyn A (2013). Food protein-induced enterocolitis syndrome (FPIES): current management strategies and review of the literature. J Allergy Clin Immunol Pract.

[39] Sicherer SH (2005). Food protein-induced enterocolitis syndrome: case presentations and management lessons. J Allergy Clin Immunol.

[40] Leonard SA, Nowak-Wegrzyn A (2011). Food protein-induced enterocolitis syndrome: an update on natural history and review of management. Ann Allergy Asthma Immunol.

[41] Boyce JA, Assa'ad A, Burks AW, Jones SM, Sampson HA, Wood RA, Plaut M, Cooper SF, Fenton MJ, Arshad SH, Bahna SL, Beck LA, Byrd-Bredbenner C, Camargo CA, Eichenfield L, Furuta GT, Hanifin JM, Jones C, Kraft M, Levy BD, Lieberman P, Luccioli S, McCall KM, Schneider LC, Simon RA, Simons FE, Teach SJ, Yawn BP, Schwaninger JM (2010). Guidelines for the diagnosis and management of food allergy in the United States: report of the NIAID-sponsored expert panel. J Allergy Clin Immunol.

[42] Simons FE, Ardusso LR, Bilo MB, El Gamal YM, Ledford DK, Ring J, Sanchez-Borges M, Senna GE, Sheikh A, Thong BY, World Allergy Organization (2011). World allergy organization guidelines for the assessment and management of anaphylaxis. World Allergy Organ J.

