# Supplementary material for: Successful prevention of extremely frequent and severe food anaphylaxis in three children by combined traditional Chinese medicine therapy
Source: Allergy Asthma Clin Immunol. 2014 Dec 20;10(1):66. doi: 10.1186/s13223-014-0066-5 (PMC4322482; doi:10.1186/s13223-014-0066-5)
Supplement: Additional file 1: — Supplemental information. [file 13223_2014_66_MOESM1_ESM.docx]

**Supplemental information**

**S. Method 1. Herbal constituents:**

**Remedy A.** *Pruni Mume* and *Phellodendri formula* (Mei Huang Tea, 0.55g/tablet) is an ethanol purified dried aqueous extract of *Prunus mume*, *Zanthoxylum schinifolium, Angelica sinensis*, *Zingiber officinalis*, *Cinnamomum cassia*, *Phellodendron chinensis*, *Panax ginseng*, and *Ganoderma lucidum* in tablet form. 12 capsules t.i.d was the full dose for patients 12 and older based on the manufacturing extraction yield of raw herbs. **Remedy B**. Fructus Jujubae Formula (Digestion tea, 0.5 g/capsule) is a dried aqueous extract of Fructus Jujubae, Endothelium Corneum Gigeriae Galli, Fructus Amomi, Rhizoma Zingiberis Recens, Radix Pseudostellariae, Rhizoma Cyperi, and Semen Coicis in capsule form (0.5g/capsule, 3 pills, b.i.d). **Remedy C.** *Phellodendron chinensis* Formula Bath Additive (Herbal Bath Additive) is a dried aqueous extract of *Cortex Phellodendri*, *Radix Rhizoma Rhei*, *Radix Sophorae Flavescentis,* *Cortex Dictamni*, *Dayscarpi Radicis*, *Fructus Tribuli Terrestris*, *and Rhizoma Smilacis Glabrae* in granule form (10g/pack). **Remedy D.** *Phellodendron chinensis* topical cream (Herbal Cream) contains 2.8% *Phellodendron chinensis* and 1% *Indigo naturalis extracts applied* after daily baths. Remedies A, C and D were produced by Chinese Academy of Traditional Chinese Medicine Sciences, Xiyuan Chinese Medicine Research and Pharmaceutical Manufacturer (Beijing, China). Remedy B was prepared by Brion Herbs Corporation (Irvine, CA). Tests for heavy metal, pesticide residues and microbial content met required standards.^(3-7)^ Patients received essentially the same treatment. Dosage adjustments were made as necessary over the course of treatment based on the individuals’ age and improvement of FSFA condition.

**S. Table 1. Definition of Severity of Reactions**

| **Symptom** | **Definitions** |
| --- | --- |
| ***SKIN SYMPTOMS*** | |
| **Itchiness** | **3 = Severe** (hard/continuous scratching → skin abrasion)  **2 = Moderate** (scratching for over 2 min at a time)  **1 = Mild** (occasional scratching)  **0 = Absent** |
| **Hives** | **3 = Severe** (widespread hives)  **2 = Moderate** (between 3 & 10 hives)  **1 = Mild** (less than 3 hives)  **0 = Absent** |
| **Swelling** | **3 = Severe** (widespread swelling)  **2 = Moderate** (noticeable lip or face swelling)  **1 = Mild** (mild lip swelling)  **0 = Absent** |
| **Rash** | **3 = Severe** (widespread redness  **2 = Moderate** (numerous red areas)  **1 = Mild** (a few areas of faint redness)  **0 = Absent** |
| ***RESPIRATORY/THROAT SYMPTOMS*** | |
| **Sneezing/ Itching** | **3 = Severe** (long bursts of sneezing, continuous nose/eye rubbing, persistent sniffing/stuffiness, eye swelling)  **2 = Moderate** (<10 bursts, intermittent nose/eye rubbing, frequent sniffing)  **1 = Mild** (rare bursts of sneezing, occasional sniffing)  **0 = Absent** |
| **Throat** | **3 = Severe** (severe tightness)  **2 = Moderate** (hoarseness, dry cough)  **1 = Mild** (<3 episodes of cough or throat cleaning; persistent throat pain/tightness)  **0 = Absent** |
| **Wheezing** | **3 = Severe** (use of chest muscles, audible wheezing)  **2 = Moderate** (wheezing upon inhalation & exhalation)  **1 = Mild** (wheezing upon exhalation; barely or inaudible)  **0 = Absent** |
| ***STOMACH/INTESTINAL SYMPTOMS*** | |
| **Stomach/ intestinal sensations** | **3 = Severe** (symptoms interfere with normal activity, severe discomfort)  **2 = Moderate** (frequent nausea/pain with normal activity)  **1 = Mild** (nausea, abdominal pain, itchy mouth/throat)  **0 = Absent** |
| **Stomach/ intestinal objective symptoms** | **3 = Severe** (>3 episodes of vomiting or diarrhea, or 2 of each)  **2 = Moderate** (2-3 episodes of vomiting/diarrhea, or 1 of each)  **1 = Mild** (1 episode of vomiting or diarrhea)  **0 = Absent** |
| ***CARDIOVASCULAR/NEUROLOGIC*** | |
| **Heart Rate (HR) & Blood Pressure (BP)**  **Neurological sings** | **3 = Severe** (cardiovascular collapse, unconscious or other signs of impaired circulation and changes in activity )  **2 = Moderate** (drop in BP, significant change in mental status)  **1 = Mild** (weak/dizzy, increased HR)  **0 = Absent** |

The criteria of symptom scores were adopted from y Sampson et al (1) with slight modifications.

Symptom scores were adopted from Sampson et al ^(1)^ modifications.

S Table 2. Quality of Life/Distress scores: Food hypersensitivity family impact (FLIP) questionnaire

S Table 2. Quality of Life/Distress scores: Food hypersensitivity family impact (FLIP) questionnaire

| **If you and your family were planning…** |
| --- |
| a **holiday/ vacation/ dinner at a restaurant**, how much would your choice be **limited** by your/your child’s food allergy? |
| to participate in **social activities involving food** (e.g. parties, holiday) how limited would your ability to participate be because of your/your child’s food allergy? |
| **How troubled have you been by…** |
| your need to spend **extra time preparing meals** (i.e. label reading, extra time shopping, preparing extra meals, etc.) due to your/your child’s food allergy? |
| your need to take **special precautions** before going out of the home (with your child) because of your/their food allergy? |
| **anxiety** relating to your/your child’s food allergy? |
| the **idea that you/your child may not overcome** your/their food allergy or by **sadness about your/your child’s burden**? |
| *(if this form is for your child)*  the possibility of (or actually) **leaving your child in the care of others**? |
| **frustration over others’ lack of appreciation** of the seriousness of food allergy? |
| *(if this form is for your child)*  your child attending **school, camp, daycare or other group activities** with children because of your child’s food allergy? |
| your **concerns for your/your child’s health** **and nutrition** because of your/their food allergy? |
| the worry that, in the event that you/your child has an **allergic reaction**, you will not be able to **resolve it**? |
| the worry that you/your child will not have a **normal life/upbringing** because of your/their food allergy? |
| issues concerning you/your child **being near others while eating** because of your/your child’s food allergy? |

Quality of Life/Distress scores were using a modified version of the “Food hypersensitivity family impact (FLIP) questionnaire (2). Key: 3 = extremely; 2 = considerably, 1 = somewhat, 0 = not at all

Quality of Life/Distress scores were using a modified version of the “Food hypersensitivity family impact (FLIP) questionnaire^(2)^. Key: 3 = extremely; 2 = considerably, 1 = somewhat, 0 = not at all

S References

(1) Sampson HA. Anaphylaxis and emergency treatment. Pediatrics 2003; 111(6 Pt 3):1601-8.

(2) Mikkelsen A, Borres MP, Bjorkelund C, Lissner L, Oxelmark L. The food hypersensitivity family impact (FLIP) questionnaire - development and first results. Pediatr Allergy Immunol 2013; 24(6):574-81.

(3) Dolan SP, Nortrup DA, Bolger PM, Capar SG. Analysis of dietary supplements for arsenic, cadmium, mercury, and lead using inductively coupled plasma mass spectrometry. J Agric Food Chem 2003; 51(5):1307-12.

(4) Caldas ED, Machado LL. Cadmium, mercury and lead in medicinal herbs in Brazil. Food Chem Toxicol 2004; 42(4):599-603.

(5) Raman P, Patino LC, Nair MG. Evaluation of metal and microbial contamination in botanical supplements. J Agric Food Chem 2004; 52(26):7822-7.

(6) The State Pharmacopoeia Commission of The People's Republic of China. Pharmacopoeia of the People's Republic of China. Version 6 ed. People's Medical Publishing House, 2005.

(7) The state of administration of Traditional Chinese Medicine of the People's Republic of China. Standards of Import and Export of Green Medicinal Plants and Their Preparations. Ministry of Foreign Trade and Economic Cooperation, People's Republic of China 2001.
